# Atypical Presentation of Pulmonary Embolism Several Months After COVID-19 Infection

**DOI:** 10.7759/cureus.12863

**Published:** 2021-01-22

**Authors:** Ayesha Jamil, Vinayasree Shyam, Karun Neupane

**Affiliations:** 1 Internal Medicine, Nazareth Hospital, Philadelphia, USA; 2 Internal Medicine, Manipal College of Medical Sciences, Pokhara, NPL

**Keywords:** pulmonary embolism (pe), covid 19, dizziness, atypical, sars-cov-2

## Abstract

A 47-year-old female with a past medical history of morbid obesity and hypertension presented with acute onset dizziness that started while she was at work one evening. She did not have chest pain or dyspnea. She had vital signs within an acceptable range, oxygen saturation of 98%-99%, and was not in acute distress. Examination including the Dix-Hallpike maneuver was unremarkable. Computed tomography angiography (CTA) of the head and neck disclosed bilateral pulmonary embolism without any evidence of cerebral ischemia. CTA chest confirmed the diagnosis of bilateral pulmonary emboli. Importantly, besides the obesity, the patient did not have any other risk factors of pulmonary embolism including recent immobilization, surgery, hormonal therapy or contraceptive use, and personal or family history of thromboembolic disorders. However, she was diagnosed with COVID-19 infection six months back with symptoms not requiring hospitalization. Following further workup for her dizziness and neurology evaluation, in the absence of any other plausible etiology, her presenting symptom was attributed to the atypical presentation of pulmonary embolism. She was treated with heparin in the hospital and discharged on apixaban. Her symptoms had resolved at the time of discharge.

## Introduction

Hypercoagulability due to coronavirus disease 2019 (COVID-19) has been a common clinical manifestation, especially in the setting of severe respiratory disease and a higher degree of inflammation. Pathogenesis is speculated to be due to this virus directly affecting all three components of Virchow's triad; endothelial injury, stasis, and activation of blood coagulation [1]. The incidence of pulmonary embolism (PE) is 2.6% to 8.9% in hospitalized COVID-19 patients and in up to one-third of COVID-19 patients in the ICU setting [2]. Here, we present an unusual case of PE with an atypical presentation in a non-hospitalized COVID-19 survivor.

## Case presentation

A 47-year-old morbidly obese, non-smoker African American female with a past medical history of hypertension and COVID-19 infection (in April 2020), presented to the emergency in late November after having a sudden episode of dizziness while at work. The patient reported that she went to work at around 11 pm and was fine till 1:30 am when she abruptly developed dizziness associated with severe nausea and three episodes of non-bilious vomiting. She described the episode as the sensation of everything in the room spinning around her. The review of the system was otherwise unremarkable. She denied a history of prolonged immobilization, recent surgery, personal or family history of blood clots, and was not taking oral contraceptives or hormonal supplements. She was a non-smoker. She denied chest pain, shortness of breath, palpitations, cough, change in vision, headache, pain in the extremities, and syncope. Of note, after testing positive for COVID-19 six months before presentation, the patient improved with supportive treatment without the need for hospitalization. She was diagnosed with systemic hypertension three years ago for which she was taking valsartan and furosemide. She had a history of knee replacement surgery in 2012 without any post-operative complications. She was a nurse by profession. There was no family history of blood clots.

**Figure 1:**
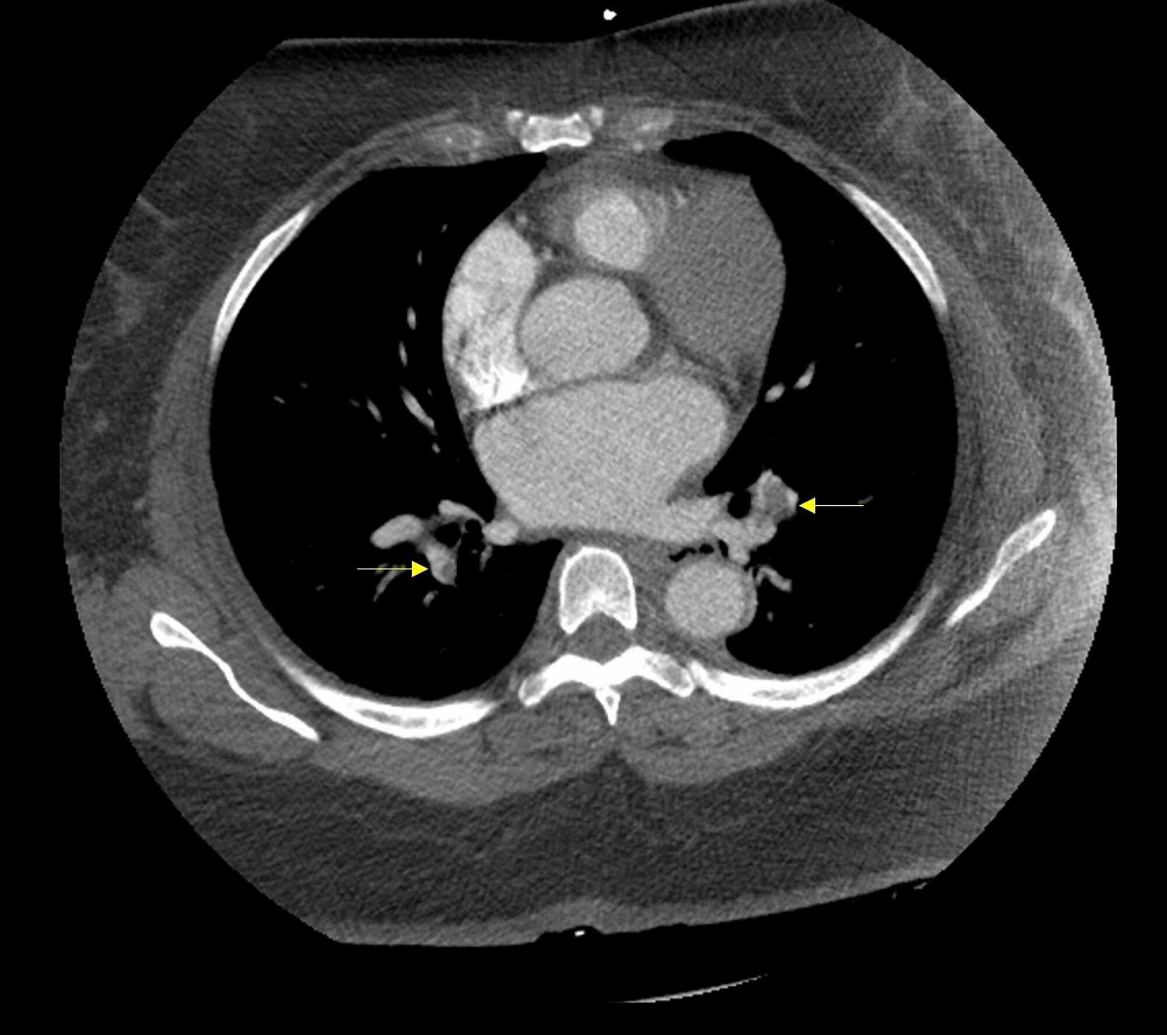
Computed tomography angiography of the chest showing bilateral pulmonary embolism (yellow arrows).

On physical examination, her blood pressure was 186/121 and her oxygen saturation was 98%-99% on room air. Other vitals were within normal limits. The patient did not have orthostatic hypotension. The Dix-Hallpike maneuver was negative. The patient’s body mass index was calculated as 46.4kg/m^2^. EKG showed sinus rhythm with a heart rate of 69 beats per minute and early progression of R-wave. Lab workup showed an increased BNP of 372 (reference range 5-125pg/ml), a slightly elevated c-reactive protein of 0.569mg/dl (reference range 0.0-0.300mg/dl), creatinine of 0.7mg/dl (reference range 0.50-1.10mg/dl), no elevation in other inflammatory markers, and troponin was also negative (done twice). The patient tested negative for COVID-19 during this visit. D-dimer was not obtained. Concerning her symptoms, computed tomography angiography (CTA) of the head and neck was done that showed bilateral pulmonary embolism without signs of cerebral ischemia. CTA chest was then ordered and showed filling defects in the right pulmonary artery extending to the lobar and segmental branches, and in lobar branches to the upper and lower lobes of the left lung, confirming bilateral PE (Figure 1).

Echocardiography showed normal filling pressures and an ejection fraction of 65%, no pulmonary hypertension, and no evidence of right ventricular strain. Given hemodynamic stability, thrombolysis/thrombectomy was not warranted by an interventional radiologist. Furthermore, based on her magnetic resonance imaging (MRI) brain findings and neurologic evaluation, no other convincing cause of her dizziness was found. During her stay, she was treated with heparin and was discharged on 5mg apixaban two tablets twice a day for one week and then one tablet twice a day for one year.

## Discussion

The common presenting features of pulmonary embolism include dyspnea, pleuritic chest pain, cough, orthopnea, calf pain, wheezing, and hemoptysis [3]. The atypical presentation of pulmonary embolism in this patient several months following non-hospitalized COVID-19 infection reveals variable disease patterns and highlights possible long-term disease complications. Thus far, cases that reported pulmonary embolism as a post-discharge complication were only up to six weeks post-discharge in hospitalized COVID-19 patients [4]. Therefore, our case highlights the need for healthcare workers to be aware of late hypercoagulable complications in less severely affected non-hospitalized severe acute respiratory syndrome coronavirus 2 (SARS-CoV-2) infected patients. It also calls attention to the possible need to lower the threshold for working up previously positive COVID-19 patients for hypercoagulable conditions.

Although the pathogenesis of hypercoagulability in COVID-19 infection has not been completely understood, the proposed mechanism can be explained as follows. Direct invasion of endothelial cells by the virus and endotheliitis and inflammation of microvasculature could play a central role in causing arteriovenous thrombosis [5]. Moreover, an increase in thrombogenic factors, such as factor VIII, not only enhances thrombin production but also induces acquired activated protein C resistance. Also, a rise in pro-inflammatory cytokines (e.g., interleukin-6) causes fibrinogen synthesis and induces tissue factor gene expression in endothelial cells and monocytes that eventually trigger thrombin generation [6]. Furthermore, elevated von-Willebrand factor results in the development of cross-linked fibrin clots, and increased fibrinogen leads to hyperviscosity. A recent study showed the formation of neutrophil extracellular traps (NET) during COVID-19 infection that originates from decondensed chromatin released to immobilize pathogens and can trigger immunothrombosis, even COVID-19 plasma triggered NET formation [7]. Besides, immobilization in critically ill and hospitalized COVID-19 patients results in stasis of blood flow which if compounded with obesity can further disrupt vascular homeostasis favoring endothelial dysfunction.

In a recent study involving more than 3,000 individuals admitted to the hospital, male sex, old age, Hispanic ethnicity, coronary artery disease, prior myocardial infarction, and high D-dimer levels at hospital presentation was marked as risk factors for venous thromboembolism (VTE) on multivariate analysis. In contrast, our patient was a middle-aged African American female with no prior history of any cardiac disease [8]. In another study at a tertiary referral center for COVID-19 patients, the incidence of acute PE was found to be 30% [9]. However, a higher incidence than reported should be considered, given not all hospitalized COVID-19 patients get CTA either due to limited resources available or to limit excessive exposure to healthcare workers. 

Since the outbreak of this pandemic, studies that are done all suggested a higher propensity of VTE in COVID-19 patients, and fortunately, some recommendations are now available regarding VTE prophylaxis in hospitalized medical, surgical, and obstetric patients. However, research data related to post-exposure DVT/PE prophylaxis in non-hospitalized COVID-19 patients are scarce and should be investigated further.

## Conclusions

Albeit several theories have been proposed explaining the pathogenesis of hypercoagulability in the setting of COVID-19 infection, the exact mechanism is still unclear. Our case highlights the importance of knowing the long term effects of COVID-19 for not only hospitalized but also for less severely affected non-hospitalized patients. The physicians should have a high index of suspicion for PE in patients with a history of prior COVID-19 infection, irrespective of timeline, disease severity, and atypical presentation.
